# A chimp algorithm based on the foraging strategy of manta rays and its application

**DOI:** 10.1371/journal.pone.0298230

**Published:** 2024-03-07

**Authors:** Guilin Yang, Liya Yu

**Affiliations:** College of Mechanical Engineering, Guizhou University, Guiyang, China; Firat Universitesi, TURKEY

## Abstract

To address the issue of poor performance in the chimp optimization (ChOA) algorithm, a new algorithm called the manta ray-based chimpa optimization algorithm (MChOA) was developed. Introducing the Latin hypercube method to construct the initial population so that the individuals of the initial population are evenly distributed in the solution space, increasing the diversity of the initial population. Introducing nonlinear convergence factors based on positive cut functions to changing the convergence of algorithms, the early survey capabilities and later development capabilities of the algorithm are balanced. The manta ray foraging strategy is introduced at the position update to make up for the defect that the algorithm is prone to local optimization, which effectively improves the optimization performance of the algorithm. To evaluate the performance of the proposed algorithm, 27 well-known test reference functions were selected for experimentation, which showed significant advantages compared to other algorithms. Finally, in order to further verify the algorithm’s applicability in actual production processes, it was applied to solve scheduling problems in three flexible workshop scenarios and an aviation engine job shop scheduling in an enterprise. This confirmed its efficacy in addressing complex real-world problems.

## 1 Introduction

As scientific research continues to advance, various practical production optimization problems are becoming more complex. Traditional optimization methods are no longer sufficient and complex optimization challenges have become a bottleneck in scientific research. In response, heuristic algorithms have been proposed as a solution. These algorithms allow the optimization of complex problems with the help of a computer. Common ones include genetic algorithms (GA) [1], simulated annealing algorithms (SA) [2], and with the technological development of science and technology, more and more population intelligence algorithms have been proposed one after another: e.g. the particle swarm optimization (PSO) algorithm, which is widely used for engineering constrained optimization problems due to its simplicity [3, 4]. Other methods, such as the Whale Optimization Algorithm (WOA), are applied to finite element model correction problems due to their fast convergence. The new Salp Swarm Algorithm (SSA) has achieved good results in the field of structural optimization [5, 6]. As well as optical heuristic algorithms are tested in constrained real engineering problems with promising results [7]. Due to the flexibility of population intelligence algorithms, there is a growing interest in developing and improving algorithms based on the characteristics of the problem. For example, To solve the spoofing detection problem, Chaos Based Optics Inspired Optimization (CBOIOs) is proposed and a large number of numerical experiments are conducted to verify the effectiveness of the algorithm [8]; An improved Arithmetic Optimization Algorithm (AOA) has been developed for the optimal design of automotive cruise control (ACC) systems [9]; Optimizing Nonlinear and Linearized Problems by Fusing the Atomic Search Optimization (ASO) Algorithm with Simulated Annealing (SA) Algorithm of hASO-SA [10]; In order to investigate the end voltage levels of fractional order proportional integral differential (FOPID) controllers, an algorithm based on a dyadic hybrid slime model with simulated annealing and Lévy flight-based reptile search and Nelder-Mead (L-RSANM) reptile search algorithms are developed [11, 12]; To construct an effective mechanism for controlling magnetically levitated objects, a skate foraging optimization algorithm integrating NM with Generalized Oppositional Learning (GOBL) is developed [13]. These applications provide a good demonstration of the diverse applications of the algorithm.

In 2020, Khishe developed a chimp optimization algorithm(ChOA) inspired by the differences in individual intelligence and abilities in the chimp population and predation activities [14]. The ChOA algorithm, compared with other optimization algorithms, has fewer parameters, is easy to implement, is relatively stable, has no gradient restrictions, and is widely used for parameter optimization of functional links [15]. However, there are still some defects such as easy premature convergence, single convergence mode, and low optimization accuracy. In response to the above problems, scholars have made different strategies to improve ChOA. Kaur et al. [16]. proposed a sine-cosine chimp optimization algorithm to reduce the slow convergence speed of the chimp algorithm and its tendency to fall into local optimality. Liu et al. [17] introduced Halton sequence and an improved nonlinear factor, combined with the idea of golden sine correlation, to propose a multi-strategy Golden Sine chimp Algorithm to improve the optimization performance of the algorithm. He et al. [18] incorporated a water wave dynamic adaptive factor into the position update process to prevent the population from converging. Wei et al. [19] introduced a hybrid time-varying continuous Levy flight strategy to enhance the algorithm’s search and exploration capabilities. Jabber et al. [20] used the conjugate gradient method to improve the algorithm and enhance the algorithm’s search accuracy.

Despite the various improvements made to the ChOA algorithm in the literature above, there has been limited in-depth research on its practical applications. Most applications have been confined to simple structural optimization problems, with a scarcity of studies on complex NP-hard problems like job shop scheduling. Furthermore, there is still room for improvement in terms of local search capability, escaping local optima, and the speed of finding the optimal solution. In light of these considerations, this paper introduces a novel chimp optimization algorithm (MChOA) based on the manta ray foraging strategy. Specifically, the MChOA algorithm utilizes a Latin hypercube method for population initialization, significantly enhancing the diversity of the initial population. It incorporates a convergence factor based on the tangent function to balance the algorithm’s search capability and improve convergence speed. Additionally, a manta ray foraging strategy is proposed for position updates to prevent local convergence and enhance the optimization performance of ChOA. Results from standard test functions and statistical analyses demonstrate the superior search performance of MChOA. The proposed algorithm is applied to solve job shop scheduling problems, conducting experiments on standard cases of 8X8, 10X10, and 10X15, as well as a production case from a specific airline. Experimental results indicate the efficiency of the proposed algorithm in solving discrete problems, offering valuable insights and references for the further development of manufacturing resource scheduling. This study also provides direction for the diversified application of the ChOA algorithm. The chapter setup of this paper is shown below: Section 2 introduces the chimpanzee optimization algorithm, section 3 presents the MChOA, section 4 conducts the corresponding experiments, section 5 introduces the application of the algorithm in job shop scheduling, and section 6 summarizes the manuscript.

## 2 chimp optimization algorithm

ChOA is inspired by the differences in intelligence and ability of individuals in chimp populations and their predatory activities. In chimp populations, chimps are divided into drivers, barriers, chasers, and attackers based on individual intelligence and ability to complete the four main steps of the hunting process: driving, barring, chasing, and attacking the prey. The mathematical formula for chimps to repel prey is shown in Eq (1), and the formula for chasing prey is shown in Eq (2).

$$\begin{array}{c} D=|cX_{P}\left(t\right)-mX_{C}\left(t\right)|\; \end{array}$$ (1)

$$\begin{array}{c} X_{C}\left(t+1\right)=X_{P}\left(t\right)-A\cdot D\; \end{array}$$ (2)

where: t denotes the current number of iterations; XP is the prey position vector, XC is the current chimp position vector, A, c, and m are the coefficient, and the equations for solving each parameter are shown in Eqs (3) and (5).

$$\begin{array}{c} A=2f\cdot r_{1}-f\; \end{array}$$ (3)

$$\begin{array}{c} c=2\cdot r_{2}\; \end{array}$$ (4)

$$\begin{array}{c} m=chaotic\_value\; \end{array}$$ (5)

where: and are random vectors between [0, 1], respectively. f is a linear convergence factor that decreases from 2.5 to 0 as the iteration proceeds. A is [-f, f] a random variable in which individual chimps approach their prey when A<1 and vice versa. m is the chaotic mapping vector; and c is the prey position influence factor on chimp expulsion and prey chasing, randomly generated between [0, 2].

After the population initialization, the positions of the attacker, barrier, driver, and chaser were selected as optimal solutions in turn, and the positions of the other chimps in the population were updated around these four chimp positions.

$$\begin{array}{c} X_{1}=X_{\text{attacker}}-A_{1}*|C_{1}*X_{\text{attacker}}-m_{1}*X|\; \end{array}$$ (6)

$$\begin{array}{c} X_{2}=X_{\text{barrier}}-A_{2}*|C_{2}*X_{\text{barrier}}-m_{2}*X|\; \end{array}$$ (7)

$$\begin{array}{c} X_{3}=X_{\text{chaser}}-A_{3}*|C_{3}*X_{\text{chaser}}-m_{3}*X|\; \end{array}$$ (8)

$$\begin{array}{c} X_{4}=X_{\text{driver}}-A_{4}*|C_{4}*X_{\text{driver}}-m_{4}*X|\; \end{array}$$ (9)

$$\begin{array}{c} X_{t+1}=\frac{\left(X_{1}+X_{2}+X_{3}+X_{4}\right)}{4} \end{array}$$ (10)

Where, Xattacker represents the position vector of the optimal solution in the population, Xbarrier represents the position vector of the second-best solution in the population, Xchaser represents the position vector of the third-best solution in the population, and Xdriver represents the position vector of the fourth-best solution in the population. X(t+1) denotes the position vector of the current chimp after the update.

## 3 Improving the chimp optimization algorithm

### 3.1 Strategies for initializing Latin hypercube populations

The quality of the iterative results is heavily influenced by the quality of the initial population [21]. However, the initial chimp algorithm generates the initial population of individuals randomly, which may not ensure diversity within the population. To address this issue, a Latin hypercube sampling method is proposed. This method is a stratified sampling technique that is based on the concept of space filling.

**Fig 1:**
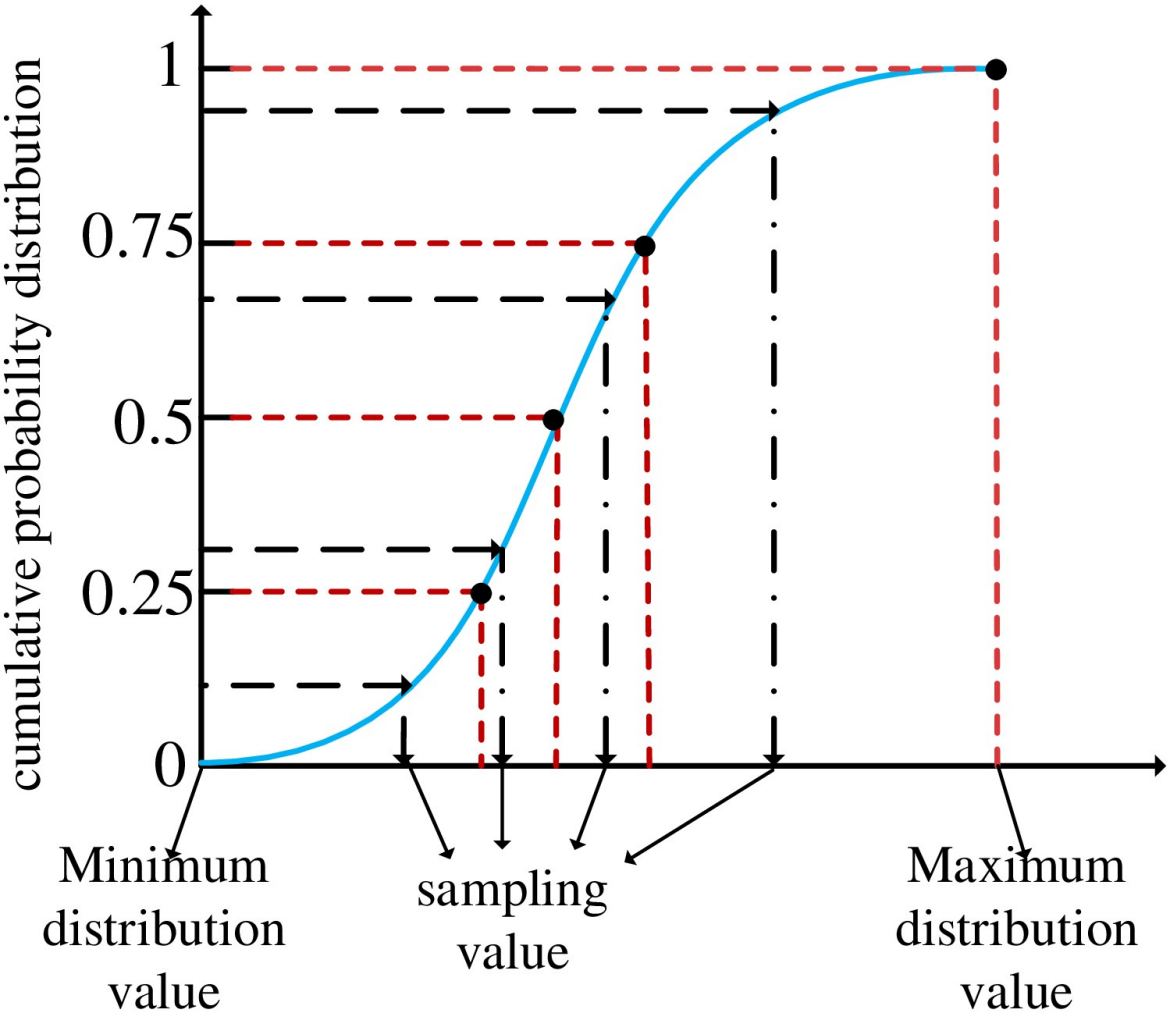
Schematic diagram of Latin hypercube sampling.

Latin Hypercube Sampling (LHS) is a multidimensional stratified sampling technique proposed by McKay et al [22]. It efficiently samples across the distribution of variables by dividing the interval [0, 1] into N equally spaced, non-overlapping subintervals. Each sub-interval is sampled independently and equally spaced to ensure that the sampling points are evenly distributed throughout the distribution. As shown in Fig 1, the cumulative curve is divided into 4 intervals. At the time of sampling, one sample is taken from each interval, and each sampling point is taken by a random mapping of values from each interval, ensuring the randomness of the initial population. The technique used in Latin Hypercube sampling is “sampling without replacement”, once a sample has been drawn from a stratum, that stratum is no longer sampled and no aggregated samples appear, thus ensuring diversity in the algorithm. The specific steps are as follows.

Step 1: Determining the size of the population: N, Dimension: dim.
Step 2: The upper and lower bounds N of the variables in each dimension are divided equally to obtain N*dim intervals of different dimensions.
Step 3: Set the random full permutation matrix M, size N*dim, each row corresponds to one population individual.
Step 4: Randomly select a value in each interval to fill the matrix M, obtain the initial population with rich diversity.

**Fig 2:**
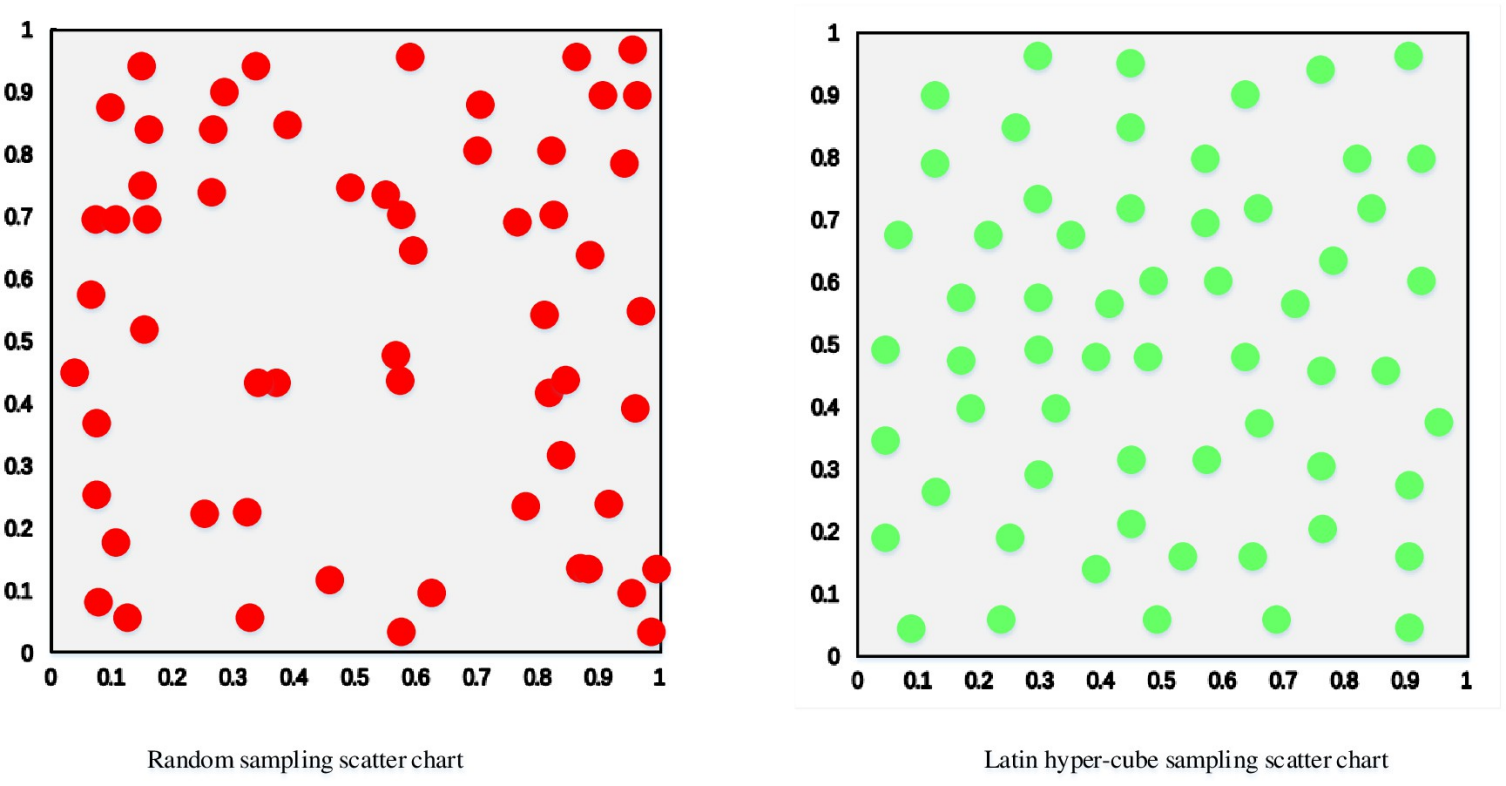
Individual distribution scatter plot.

Fig 2 depicts a comparison between a randomly generated scatter plot of population individuals and a scatter plot generated using the Latin hypercube population initialization method. The plot is generated with parameters set to N = 40 and dim = 2. It is evident that the Latin hypercube method produces an initial population that is more evenly distributed in the solution space and does not contain overlapping individuals. This results in a more diverse population than the randomly generated initial population and can prevent the algorithm’s search results from being distorted to some extent.

### 3.2 Nonlinear convergence factors

The basic ChOA algorithm uses a convergence factor f that decreases linearly with the number of iterations. However, this strategy does not balance the global search ability and local exploitation ability of the algorithm well, and it does not account for the actual search process, resulting in poor search performance. To address this issue, we propose a nonlinear convergence factor based on the tangent function, which decays slower in the early stage to enhance global search ability and faster in the later stage to enhance local exploitation ability. We also introduce a convergence factor adjustment factor to control the convergence magnitude. The formula for the nonlinear convergence factor based on the tangent function is presented in Eq (11).

$$\begin{array}{c} f=f_{initial}*\left(1-\text{tan}\left(\frac{1}{\varepsilon }*\frac{t}{T_{\text{max}}}\in \pi \right)\right)\; \end{array}$$ (11)

where t represents the current iteration number, finitial is the initial value of the convergence factor, Tmax is the maximum number of iterations, it can be observed that the convergence factor f varies non-linearly as the number of iterations increases. This approach helps to balance the algorithm’s ability to explore the global solution space and exploit local search areas.

### 3.3 Foraging strategies of manta rays

This paper is based on inspiration from previous literature [23]. In order to compensate for the premature convergence tendency of the basic ChOA algorithm, this paper introduces a foraging strategy for manta rays, which utilizes a unique foraging method distinct from that of other group animals. The position of the food source is considered as a tumbling pivot point, and for each hunt, the manta ray tumbles towards a mirror image of the prey center.

$$\begin{array}{c} x_{i}^{dim}\left(t+1\right)=x_{i}^{dim}\left(t\right)+2\cdot \left(R_{1}x_{best}^{dim}-R_{2}x_{i}^{dim}\left(t\right)\right)i=1,\cdots ,N\; \end{array}$$ (12)

where is the prey location; N is the number of manta rays in the group. R1 and R2 is the random number of [0, 1].

**Fig 3:**
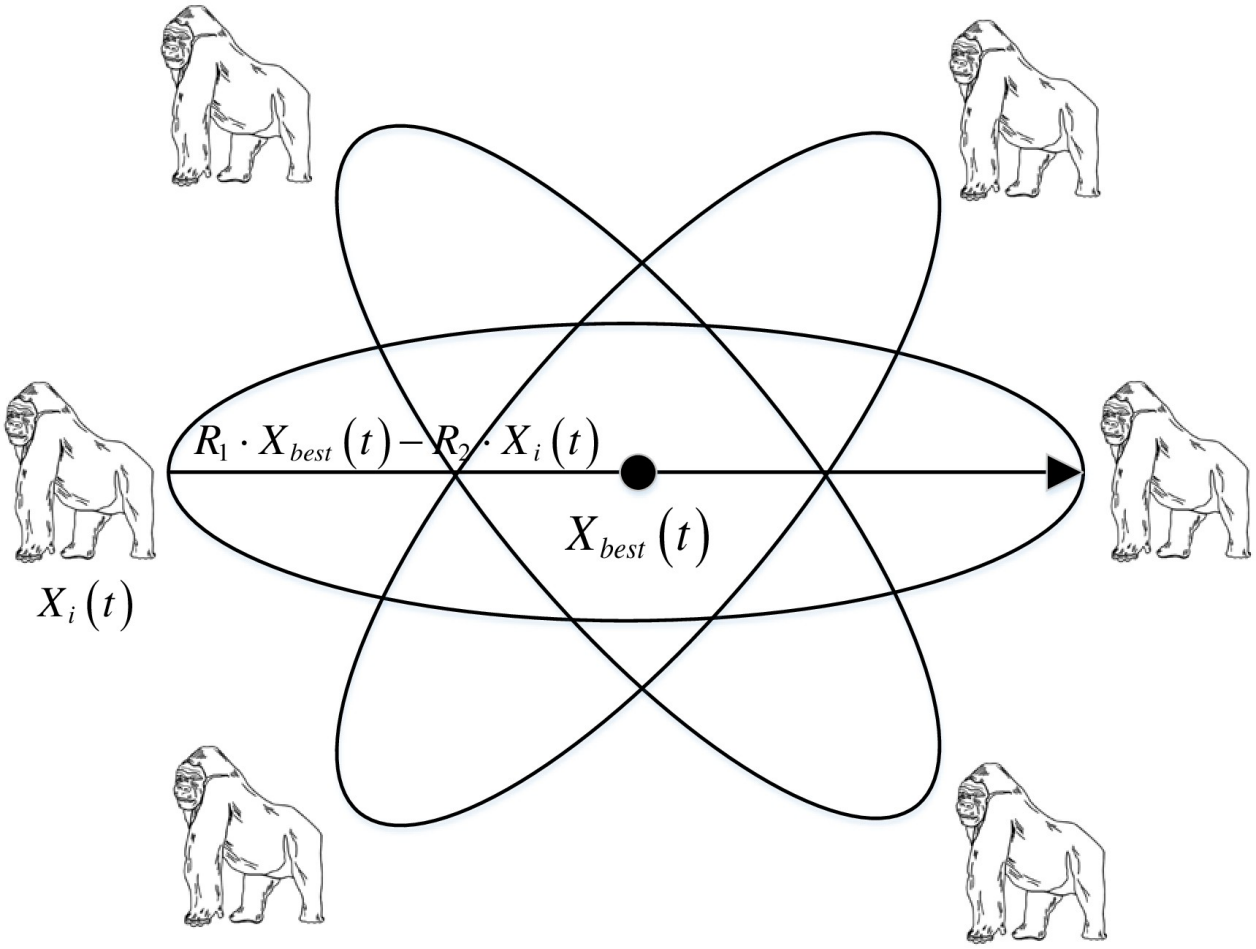
Manta ray foraging strategies.

The MChOA algorithm introduced in this paper incorporates the manta foraging strategy, which is inspired by the literature [0], to address the premature convergence issue that may occur in the basic ChOA algorithm. The foraging strategy is unique compared to other group animals, and it involves the chimps using the manta foraging method to move between their current position and a mirror position centered on the prey. This causes the distance between the individual and the prey to decrease while reducing the fluctuation in position. With each iteration, the search space envelopes become smaller, and each chimp moves closer to the prey. During the iteration, the current individual is compared to the chimp at the mirror position in terms of fitness, and if the current position is less fit, it will be replaced by the chimp at the mirror position. As the iterations progress, the algorithm becomes more likely to jump out of the local optimum. This manta foraging strategy differs from classical backward learning as it is centered around the chimp with the lowest fitness, which enhances the algorithm’s convergence. Fig 3 provides a schematic representation of the manta ray foraging strategy.

### 3.4 MChOA algorithm steps

**Fig 4:**
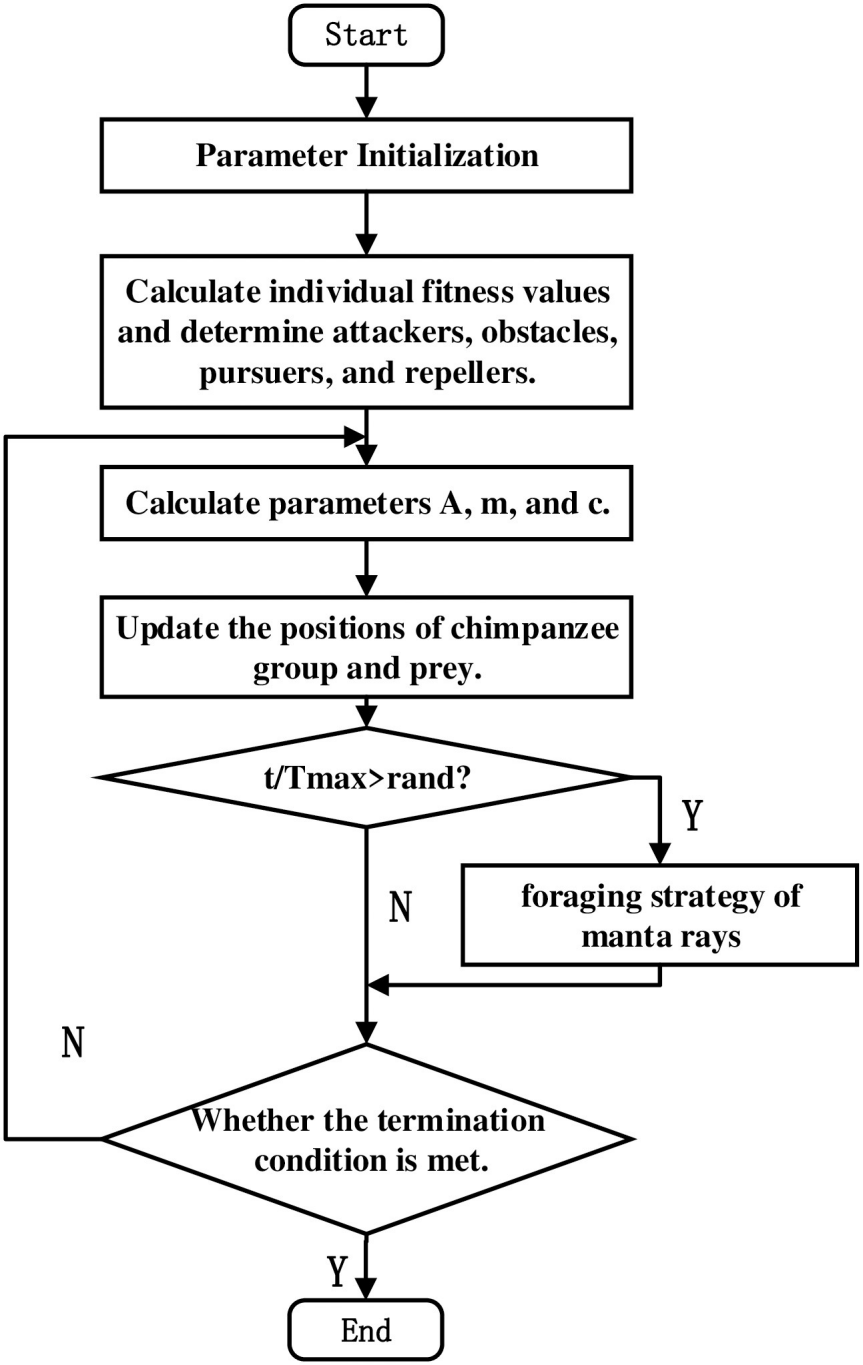
MChOA algorithm flow chart.

Algorithm 1 is the pseudo-code for the MChOA algorithm that incorporates the improved strategies mentioned above. The algorithm flowchart is shown in Fig 4:

**Algorithm 1 Framework of MChOA**

1: Initialization parameters

2: Latin square cube strategy for initializing populations

3: Calculate all individual fitness values and rank them to determine *X*_*attacker*_, *X*_*barrier*_, *X*_*chaser*_, *X*_*driver*_

4: **while** *t* < *T*_*max*_ **do**

5:  **for** *i* = 1 to *N* **do**

6:   Calculate the value of the non-linear convergence factor *f* according to Eq (11).

7:   Calculate the values of the parameters *A*, *m*, *c* according to Eqs (3, 4 and 5)

8:   Update individual positions according to Eq (10)

9:   **if** $\frac{t}{T_{max}}>\text{rand}$ **then**

10:    Update individual positions according to Eq (12)

11:   **end if**

12:  **end for**

13:  Update the values of parameters *A*, *m*, *c*

14:  Calculate the fitness of all individuals

15:  Update *X*_*attacker*_, *X*_*barrier*_, *X*_*chaser*_, *X*_*driver*_

16:  *t* = *t* + 1

17: **end while**

18: Return to *X*_*attacker*_

19: End

## 4 Simulation experiment and result analysis

### 4.1 Experimental design and test functions

In the experimental part of this paper, 27 benchmark test functions have been selected to verify the performance of the algorithm, where F24-F27 are the CEC2020 benchmark functions, which have been tested in many literatures and are given in Table 1, where dim denotes its dimension, range denotes its definition domain, and fmin is the theoretical optimal value. The number of populations was set to100 and the number of iterations (maximum) Tmax was set to 500. 30 simulations were performed in the same operating environment and the mean and standard deviation of each test function were recorded. The criteria were: the smaller the mean value, the better the search performance, and in the special case of equal means, the smaller the standard deviation, the less fluctuation in the search, and the more stable the algorithm.

**Table 1:** Benchmarking functions.

ID	Function	Dim	Range	*f*_*min*_
*F*1	$F_{1}(x)=\sum_{i=1}^{n}x_{i}^{2}$	30/50/100	[-100,100]	0
*F*2	$F_{2}(x)=\sum_{i=1}^{n}|x_{i}|+\prod_{i=1}^{n}|x_{i}|$	30/50/100	[-10,10]	0
*F*3	$F_{3}(x)=\sum_{i=1}^{n}{\left(\sum_{j-1}^{i}\sum_{i=1}^{n}\right)}^{2}$	30/50/100	[-100,100]	0
*F*4	*F*_4_(*x*) = *max*_*i*_{|*x*_*i*_|, 1≤*i*≤*n*}	30/50/100	[-100,100]	0
*F*5	$F_{5}(x)=\sum_{i=1}^{n}[[100{\left(x_{i+1}-x_{i}^{2}\right)}^{2}]+{\left(x_{i}-1\right)}^{2}]$	30/50/100	[-30,30]	0
*F*6	$F_{6}(x)=\sum_{i=1}^{n}{\left([x_{i}+0.5]\right)}^{2}$	30/50/100	[-100,100]	0
*F*7	$F_{7}(x)=\sum_{i=1}^{n}i{x_{i}}^{4}+random[0,1]$	30/50/100	[-1.28,1.28]	0
*F*8	$F_{8}(x)=\sum_{i=1}^{n}-x_{i}\text{sin}(\sqrt{\left|x_{i}\right|})$	30/50/100	[-500,500]	-418*d
*F*9	$F_{9}(x)=\sum_{i=1}^{n}[x_{i}^{2}-10\text{cos}(2\pi x_{i})+10]$	30/50/100	[-5.12,5.12]	0
*F*10	$F_{10}(x)=-20\text{exp}(-0.2\sqrt{\frac{1}{n}\sum_{i=1}^{n}x_{i}^{2}})-\text{exp}(\frac{1}{n}\sum_{i=1}^{n}\text{cos}(2\pi x_{i}))$	30/50/100	[-32,32]	0
*F*11	$F_{11}(x)=\frac{1}{4000}\sum_{i=1}^{n}x_{i}^{2}-\prod_{i=1}^{n}\text{cos}(\frac{x_{i}}{\sqrt{i}})\;\;\;+1$	30/50/100	[-600,600]	0
*F*12	$F_{12}(x)=\frac{\pi }{n}\{10\text{sin}(\pi y_{1})+\sum_{i=1}^{n-1}{\left(y_{i}-1\right)}^{2}[1+10\text{si}n^{2}(\pi y_{i+1})]+{\left(y_{n}-1\right)}^{2}\}+\sum_{i=1}^{n}u(x_{i},10,100,4)$	30/50/100	[-50,50]	0
*F*13	$F_{13}(x)=0.1\{\text{si}n^{2}(3\pi x_{1})+\sum_{i=1}^{n}{\left(x_{i}-1\right)}^{2}[1+\text{si}n^{2}(3\pi x_{i}+1)]+{\left(x_{n}-1\right)}^{2}[1+\text{si}n^{2}(1\pi x_{n})]\}+\sum_{i=1}^{n}u(x_{i},5,100,4)$	30/50/100	[-50,50]	0
*F*14	$F_{14}(x)={\left(\frac{1}{500}+\sum_{j=1}^{25}\frac{1}{j+\sum_{i=1}^{2}{\left(x_{i}-a_{ij}\right)}^{6}}\right)}^{-1}$	2	[-65,65]	1
*F*15	$F_{15}(x)=\sum_{i=1}^{11}{\left[a_{i}-\frac{x_{1}({b_{i}}^{2}+b_{i}x_{2})}{{b_{i}}^{2}+b_{i}x_{3}+x_{4}}\right]}^{2}$	4	[-5,5]	0.0003
*F*16	$F_{16}(x)=4x_{1}^{2}-2.1x_{1}^{4}+\frac{1}{3}x_{1}^{6}+x_{1}x_{2}-4x_{2}^{2}+4x_{2}^{4}$	2	[-5,5]	-1.0316
*F*17	$F_{17}(x)={\left(x_{2}-\frac{5.1}{4\pi^{2}}x_{1}^{2}+\frac{5}{\pi }x_{1}-6\right)}^{2}+10(1-\frac{1}{8\pi })\text{cos}x_{1}+10$	2	[-5,5]	0.398
*F*18	$F_{18}(x)=\sum_{i=1}^{11}{\left[a_{i}-\frac{x_{1}({b_{i}}^{2}+b_{i}x_{2})}{{b_{i}}^{2}+b_{i}x_{3}+x_{4}}\right]}^{2}$	2	[-2,2]	3
*F*19	$F_{19}(x)=-\sum_{i=1}^{4}c_{i}\text{exp}(-\sum_{j=1}^{3}a_{\text{ij}}{\left(x_{j}-p_{\text{ij}}\right)}^{2})$	3	[0, 1]	-3.86
*F*20	$F_{20}(x)=-\sum_{i=1}^{4}c_{i}\text{exp}(-\sum_{j=1}^{6}a_{\text{ij}}{\left(x_{j}-p_{\text{ij}}\right)}^{2})$	6	[0, 1]	-3.32
*F*21	$F_{21}(x)=-\sum_{i=1}^{7}{\left[(X-b_{i}){\left(X-b_{i}\right)}^{T}+C_{i}\right]}^{-1}$	4	[0, 10]	-10.1532
*F*22	$F_{22}(x)=-\sum_{i=1}^{1}0{\left[(X-b_{i}){\left(X-b_{i}\right)}^{T}+C_{i}\right]}^{-1}$	4	[0, 10]	-10.4028
*F*23	$F_{23}(x)=-\sum_{i=1}^{5}{\left[(X-b_{i}){\left(X-b_{i}\right)}^{T}+C_{i}\right]}^{-1}$	4	[0, 1]	-10.5363
*F*24	$F_{24}(x)=ExpandedRosenbrock’sPlusGriewangk’sFunction$	10	-	1900
*F*25	*F*_25_(*x*) = *HybridFunction*1	10	-	1700
*F*26	*F*_26_(*x*) = *HybridFunction*2	10	-	1600
*F*27	*F*_27_(*x*) = *CompositionFunction*1	10	-	2200

### 4.2 Comparison of optimization results for each algorithm test function

#### 4.2.1 Parameter setting

In order to validate the performance of the proposed algorithms, we have selected a number of well-established algorithms with a wide variety of performance characteristics in exploration and exploitation that have been tested with the same benchmark functions, including: ChOA, AGWO [24], TACPSO [25], MPSO [26], MPA [27], NGO [28].

The Table 2 gives the parameter settings of each algorithm, where it should be noted that the above algorithm parameters are set according to those in Ref.

**Table 2:** Algorithm parameters.

Algorithms	Parameters
ChOA	*r*_1_, *r*_2_ = rand(0, 1), *f* ∈ (0, 2.5), *u* ∈ (0, 2.5)
AGWO	*B* = 0.8, *a* = 2
TACPSO	*c*_1_, *c*_2_ = 2, *w*_max_ = 0.9, *w*_min_ = 0.2
MPSO	*c*_1_, *c*_2_ = 2, *w*_max_ = 0.9, *w*_min_ = 0.2
MPA	*F*_*ADs*_ = 0.2, *P* = 0.5, *CF* = 1
NGO	Leader position update probability = 0.5
MChOA	*r*_1_, *r*_2_ = rand(0, 1), *f* ∈ (0, 2.5), *u* ∈ (0, 2.5), *S* = 2

#### 4.2.2 Solving results on F1-F13

The single-peak test function as well as the multi-peak test function can evaluate the survey and development capability of the algorithm. To verify the performance of the algorithm in different dimensions, this section sets the test function dimensions as 30, 50, and 100, respectively, and the solution is shown in Tables 3–5. The mean values obtained by MChOA in solving the single-peak test function as well as the multi-peak test function (F1-F13) are mostly better than other algorithms. In addition, the proposed algorithm shows good stability, and the proposed algorithm performs more consistently on most of the test functions in terms of the standard deviation criterion. Overall, MChOA shows significant advantages in terms of optimization accuracy, stability and robustness.

**Table 3:** Experimental results of F1-F13(dim = 30).

		MChOA	ChOA	AGWO	TACPSO	MPSO	MPA	NGO
F1	Average	**0.00E+00**	7.11E-11	1.43E-184	8.86E-05	6.26E-04	3.04E-23	1.36E-89
	std	**0.00E+00**	2.25E-10	1.69E-111	2.76E-04	1.76E-03	2.63E-23	1.24E-89
F2	Average	**2.59E-173**	1.17E-07	3.76E-101	1.42E-02	1.11E+01	1.69E-13	6.00E-46
	std	**3.74E-173**	3.18E-07	5.48E-101	2.50E-02	7.35E+00	1.64E-13	3.76E-46
F3	Average	**7.14E-252**	1.21E+00	1.86E-99	9.63E+01	6.74E+03	3.85E-06	6.81E-22
	std	**1.96E-253**	1.45E+00	2.86E-99	2.05E+02	4.34E+03	4.40E-06	1.50E-21
F4	Average	**1.34E-159**	1.10E-02	7.31E-74	1.47E+00	3.84E+00	1.87E-09	3.85E-38
	std	**2.32E-159**	1.02E-02	1.98E-73	5.07E-01	1.58E+00	7.73E-10	1.89E-38
F5	Average	2.70E+01	2.90E+01	2.73E+01	5.48E+01	1.85E+04	**2.36E+01**	2.50E+01
	std	3.56E-01	**1.09E-02**	2.78E-01	4.16E+01	3.77E+04	3.60E-01	3.92E-01
F6	Average	2.93E-01	9.28E-01	2.49E+00	4.30E-06	5.70E-04	**4.50E-09**	4.40E-07
	std	5.21E-02	2.77E-01	5.61E-01	3.94E-06	1.68E-03	**6.33E-10**	8.83E-08
F7	Average	**2.60E-05**	3.09E-04	6.02E-05	2.02E-02	2.94E-01	7.56E-04	3.78E-04
	std	**1.49E-05**	1.32E-04	4.79E-05	7.15E-03	8.48E-01	2.89E-04	1.64E-04
F8	Average	**-1.25E+04**	-5.78E+03	-3.58E+03	-9.72E+03	-8.83E+03	-9.65E+03	-8.09E+03
	std	1.25E+02	**1.03E+02**	4.19E+02	5.88E+02	7.32E+02	4.69E+02	3.69E+02
F9	Average	**0.00E+00**	1.04E+02	0.00E+00	5.42E+01	9.61E+01	0.00E+00	0.00E+00
	std	**0.00E+00**	2.23E+01	0.00E+00	1.42E+01	3.33E+01	0.00E+00	5.06E-15
F10	Average	**4.44E-16**	2.00E+01	4.00E-15	5.32E-01	8.33E-01	1.05E-12	5.06E-15
	std	**0.00E+00**	1.38E-03	0.00E+00	6.95E-01	9.82E-01	3.85E-13	1.72E-15
F11	Average	**0.00E+00**	2.80E-03	0.00E+00	3.13E-07	2.74E-06	0.00E+00	0.00E+00
	std	**0.00E+00**	4.55E-03	0.00E+00	9.88E-03	2.49E-02	0.00E+00	0.00E+00
F12	Average	1.37E-02	7.89E-02	1.39E-01	3.34E-01	3.44E-01	**4.35E-10**	6.53E-08
	std	4.82E-03	6.15E-02	4.61E-02	3.34E-01	3.86E-01	**2.71E-10**	1.91E-08
F13	Average	2.78E-01	3.11E+00	1.80E+00	8.82E-03	8.71E-03	**5.12E-09**	2.18E-02
	std	4.57E-02	1.25E-01	1.63E-01	1.35E-02	9.51E-03	**2.00E-09**	1.64E-02

**Table 4:** Experimental results of F1-F13(dim = 50).

		MChOA	ChOA	AGWO	TACPSO	MPSO	MPA	NGO
F1	Average	**0.00E+00**	4.82E-07	1.11E-150	2.06E+00	5.47E+00	2.19E-21	1.35E-86
	std	**0.00E+00**	9.95E-07	1.63E-150	2.70E+00	1.24E+01	1.51E-21	1.47E-86
F2	Average	**9.61E-163**	3.60E-06	2.43E-84	1.86E+00	53.9186	2.45E-12	1.37E-44
	std	**1.63E-163**	9.34E-06	3.22E-84	2.96E+00	1.43E+01	1.58E-12	1.04E-44
F3	Average	**3.85E-244**	365.9423,	1.36E-86	4.54E+03	3.61E+04	2.64E-03	2.09E-16
	std	**3.56E-233**	1.86E+02	4.09E-86	3.62E+03	1.20E+04	2.47E-03	2.94E-16
F4	Average	**5.17E-157**	1.33E+00	3.52E-64	1.59E+01	3.01E+01	1.82E-08	5.50E-36
	std	**1.08E-156**	1.45E+00	8.29E-64	3.64E+00	4.35E+00	5.39E-09	2.37E-36
F5	Average	4.83E+01	4.89E+02	4.75E+01	9.28E+03	1.92E+04	**4.45E+01**	4.56E+01
	std	4.01E-01	6.98E-01	4.28E-01	2.84E+04	3.75E+04	**3.83E-01**	4.62E-01
F6	Average	9.19E-01	2.46E+00	6.05E+00	4.00E-01	1.02E+03	**9.58E-06**	1.10E-02
	std	1.79E-01	5.19E-01	3.02E-01	2.83E-01	3.19E+03	**1.85E-05**	4.29E-03
F7	Average	3.73E-05	1.11E-03	1.06E-04	9.48E-02	2.86E+00	1.09E-03	4.78E-04
	std	3.27E-05	2.06E-03	8.50E-05	5.53E-02	3.33E+00	4.21E-04	1.62E-04
F8	Average	**-2.06E+04**	-9.33E+03	-4.33E+03	-1.43E+04	-1.37E+04	-1.56E+04	-1.14E+04
	std	**3.91E+02**	5.41E+01	4.80E+02	1.02E+03	8.07E+02	7.27E+02	3.83E+02
F9	Average	**0.00E+00**	2.37E+02	0.00E+00	1.36E+02	2.82E+02	0.00E+00	0.00E+00
	std	**0.00E+00**	3.69E+01	0.00E+00	3.49E+01	4.44E+01	0.00E+00	0.00E+00
F10	Average	**4.44E-16**	2.00E+01	5.42E-15	2.48E+00	4.84E+00	7.15E-12	5.06E-15
	std	**0.00E+00**	8.33E-04	1.83E-15	7.66E-01	4.20E+00	2.86E-12	1.72E-15
F11	Average	**0.00E+00**	8.87E-03	0.00E+00	3.59E-01	9.91E+00	0.00E+00	0.00E+00
	std	**0.00E+00**	7.83E-03	0.00E+00	3.32E-01	2.86E+01	0.00E+00	0.00E+00
F12	Average	**4.11E-02**	1.41E+01	9.71E-01	5.65E+07	1.74E+09	1.49E-01	3.71E-01
	std	1.09E-02	3.77E+01	**3.14E-02**	2.22E+07	3.64E+08	1.27E-02	1.81E-02
F13	Average	5.45E-01	4.98E+00	3.80E+00	1.55E+01	2.60E+01	**7.30E-03**	3.71E-01
	std	1.42E-01	2.28E-01	1.83E-01	1.01E+01	1.47E+01	**8.50E-03**	1.27E-01

**Table 5:** Experimental results of F1-F13(dim = 100).

		MChOA	ChOA	AGWO	TACPSO	MPSO	MPA	NGO
F1	Average	**4.84E-322**	2.35E-03	4.95E-123	1.10E+03	1.66E+04	4.27E-19	2.76E-84
	std	**3.21E-319**	3.45E-03	8.15E-123	4.91E+02	6.48E+03	3.77E-19	2.60E-84
F2	Average	**4.05E-166**	2.13E-04	2.49E-68	6.30E+01	2.40E+02	3.29E-11	1.61E-43
	std	**3.45E-150**	3.39E-04	2.03E-68	2.65E+01	5.67E+01	2.54E-11	6.24E-44
F3	Average	**2.59E-234**	2.86E+04	5.00E-69	5.36E+04	1.74E+05	2.11E+00	1.85E-08
	std	**3.24E-234**	1.56E+04	1.58E-68	1.77E+04	5.01E+04	2.52E+00	5.85E-08
F4	Average	**2.09E-150**	2.72E+01	2.53E-55	3.87E+01	2.42E-07	5.52E+01	4.86E-34
	std	**6.34E-150**	1.86E+01	4.52E-55	3.86E+00	7.49E-08	4.85E+00	1.83E-34
F5	Average	9.81E+01	1.02E+02	9.79E+01	3.77E+05	9.10E+06	**9.57E+01**	9.64E+01
	std	**4.10E-02**	2.72E+00	5.55E-01	2.92E+05	2.52E+07	8.84E-01	7.95E-01
F6	Average	1.02E+00	2.53E+00	6.25E+00	1.55E+00	4.59E+00	**3.20E-06**	1.01E-02
	std	1.47E-01	2.32E-01	2.18E-01	1.68E+00	6.83E+00	**7.47E-06**	4.78E-03
F7	Average	**4.11E-05**	4.34E-03	1.74E-04	1.24E+00	9.41E+01	1.13E-03	5.27E-04
	std	**3.55E-05**	3.91E-03	1.29E-04	4.95E-01	7.60E+01	4.96E-04	1.75E-04
F8	Average	**-4.11E+04**	-1.82E+04	-5.99E+03	-2.48E+04	-2.41E+04	-2.82E+04	-1.87E+04
	std	**1.64E+02**	7.72E+01	6.51E+02	1851.759	1.51E+03	8.07E+02	9.84E+02
F9	Average	**0.00E+00**	1.21E+02	0.00E+00	3.53E+02	6.83E+02	0.00E+00	0.00E+00
	std	**0.00E+00**	1.28E+02	0.00E+00	4.65E+01	7.96E+01	0.00E+00	0.00E+00
F10	Average	**4.44E-16**	2.00E+01	6.84E-15	8.12E+00	6.24E-11	1.65E+01	7.19E-15
	std	**0.00E+00**	3.80E-03	1.50E-15	1.58E+00	2.64E-11	1.35E+00	1.12E-15
F11	Average	**0.00E+00**	2.82E-02	0.00E+00	1.08E+01	1.39E+02	0.00E+00	0.00E+00
	std	**0.00E+00**	4.59E-02	0.00E+00	6.76E+00	4.92E+01	0.00E+00	0.00E+00
F12	Average	3.09E-02	5.17E-01	6.20E-01	2.74E+01	1.88E+04	**1.12E-02**	3.04E-02
	std	7.20E-03	2.33E-01	2.77E-02	1.10E+01	4.41E+04	**3.80E-03**	5.83E-03
F13	Average	**1.85E+00**	9.79E+00	8.98E+00	7.46E+04	4.13E+07	3.92E+00	4.67E+00
	std	5.80E-01	**1.60E-01**	1.42E-01	1.57E+05	1.30E+08	3.10E+00	1.89E+00

**Fig 5:**
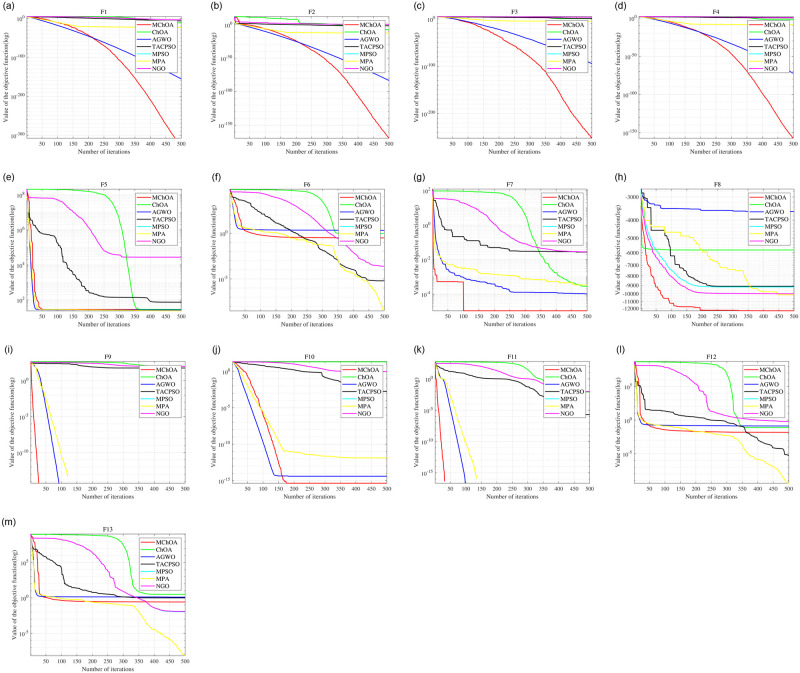
Function convergence curve.

To further demonstrate the superiority of the MChOA algorithm, the convergence curves of each algorithm are shown in Fig 5 for the sake of brevity. It can be seen that although the MChOA algorithm does not show a significant convergence speed advantage in the early stage, its advantage gradually emerges in the later stages of the optimization search process. This can be attributed to the convergence factor based on the tangent function, which balances the global search ability and local availability of the algorithm. Regarding the optimization results, MChOA has several orders of magnitude lead over other algorithms in most of the benchmark functions, which is attributed to the improvements at the population initialization and the position update in this paper. In summary, the MChOA algorithm has a large improvement in the search performance.

#### 4.2.3 Solving results on F14-F27

F14-F27 are multimodal functions with fixed dimensions, and they are not analyzed in different dimensions because their dimensions are determined. Since the experimental results do not vary much, many algorithms can obtain optimal solutions on multimodal functions of fixed dimensions without marking the optimal values, and the comparison between the algorithm proposed in this paper and other algorithms through the results shown in Table 6 shows that the algorithm proposed in this paper achieves a good balance between the exploration and development stages.

**Table 6:** Experimental results of F14-F27.

		MChOA	ChOA	AGWO	TACPSO	MPSO	MPA	NGO
F14	Average	9.98E-01	9.98E-01	4.14E+00	9.98E-01	9.98E-01	9.98E-01	9.98E-01
	std	4.85E-10	4.56E-07	3.58E+00	1.28E-16	7.40E-17	0.00E+00	0.00E+00
F15	Average	7.34E-04	1.25E-03	2.35E-03	3.99E-04	3.07E-04	2.50E-03	3.07E-04
	std	2.53E-04	1.16E-05	6.33E-03	2.90E-04	1.79E-15	6.28E-03	7.55E-10
F16	Average	-1.03E+00	-1.03E+00	-1.03E+00	-1.03E+00	-1.03E+00	-1.03E+00	-1.03E+00
	std	3.79E-07	7.42E-07	2.80E-08	7.40E-17	2.34E-16	7.40E-17	0.00E+00
F17	Average	3.98E-01	3.98E-01	3.98E-01	3.98E-01	3.98E-01	3.98E-01	3.98E-01
	std	2.43E-05	1.01E-04	2.59E-06	0.00E+00	0.00E+00	0.00E+00	0.00E+00
F18	Average	3.00E+00	3.00E+00	3.00E+00	3.00E+00	3.00E+00	3.00E+00	3.00E+00
	std	2.17E-05	1.07E-05	1.67E-07	1.38E-15	1.47E-15	1.44E-15	5.13E-16
F19	Average	-3.86E+00	-3.86E+00	-3.86E+00	-3.86E+00	-3.86E+00	-3.86E+00	-3.86E+00
	std	3.75E-02	2.44E-03	8.52E-05	8.63E-16	4.68E-16	9.36E-16	9.36E-16
F20	Average	-3.32E+00	-2.71E+00	-3.24E+00	-3.24E+00	-3.32E+00	-3.28E+00	-3.32E+00
	std	9.04E-02	3.76E-01	6.82E-02	5.74E-02	1.48E-12	7.07E-02	4.68E-16
F21	Average	-5.05E+00	-4.20E+00	-8.63E+00	-8.13E+00	-1.02E+01	-6.38E+00	-1.02E+01
	std	2.23E-02	1.75E+00	2.44E+00	2.61E+00	2.23E-11	3.40E+00	4.82E-14
F22	Average	-6.26E+00	-4.64E+00	-8.57E+00	-9.64E+00	-1.04E+01	-8.0556,	-1.04E+01
	std	2.87E+00	-8.57E+00	3.00E+00	2.42E+00	1.15E-11	3.11E+00	1.78E-15
F23	Average	-1.05E+01	-5.48E+00	-1.05E+01	-9.23E+00	-1.05E+01	-1.05E+01	-1.05E+01
	std	1.57E-15	1.18E+00	4.06E-03	2.80E+00	1.57E-15	6.43E-12	1.57E-15
F24	Average	1.90E+03	4.87E+04	1.90E+03	1.45E+04	5.32E+04	1.90E+03	1.90E+03
	std	2.20E+03	1.18E+04	4.06E+03	2.80E+04	1.57E+04	6.43E+03	1.57E+03
F25	Average	5.06E+03	8.21E+04	2.71E+04	1.74E+05	2.73E+04	5.07E+03	5.75E+03
	std	3.78E+03	7.45E+04	9.45E+03	6.78E+05	4.56E+04	9.78E+03	8.45E+03
F26	Average	1.06E+03	1.12E+03	1.06E+03	4.32E+04	8.75E+03	1.06E+03	1.06E+03
	std	4.69E-08	1.48E-06	3.78E+-08	7.63E+00	3.97E+01	2.45E-12	3.78E-09
F27	Average	2.30E+03	2.80+03	2.30E+03	2.53E+03	2.90E+03	2.30E+03	2.30E+03
	std	8.09E-11	3.12E-01	4.63–03	7.62E+00	4.97E-05	8.41E-10	1.32E-09

### 4.3 Statistical analysis

To verify the difference between the MChOA algorithm and the other algorithms, we used the Wilcoxon rank sum test to calculate the p-value to compare the difference between the algorithms. If p<0.05, it means that there is a significant difference between the two algorithms, #DIV/0 means that the algorithm gets the optimal value every time and cannot be compared, Table 7 shows that the p-values of Wilcoxon rank sum test for MChOA are basically less than 5%, which indicates that statistically speaking, the advantage of MChOA for basic function finding is obvious, thus further demonstrating the robustness of MChOA.

**Table 7:** Wilcoxon rank sum test results.

	ChOA	AGWO	TACPSO	MPSO	MPA	NGO
F1	3.31E-20	3.31E-20	3.31E-20	3.31E-20	3.31E-20	3.31E-20
F2	2.83E-10	2.84E-05	3.78E-18	4.13E-17	1.30E-03	4.45E-09
F3	3.31E-20	3.31E-20	3.31E-20	3.31E-20	3.31E-20	3.31E-20
F4	3.06E-18	4.25E-04	3.75E-06	4.32E-15	4.35E-10	9.36E-07
F5	5.45E-15	3.75E-09	4.12E-11	8.45E-15	4.36E-12	1.31E-10
F6	8.45E-10	4.32E-01	5.21E-07	6.12E-09	1.12E-03	3.78E-08
F7	1.54E-07	3.65E-04	7.32E-04	9.45E-06	5.64E-04	9.12E-03
F8	7.06E-18	7.21E-09	7.06E-18	7.06E-18	6.45E-03	9.21E-10
F9	3.31E-20	#DIV/0	3.31E-20	3.31E-20	#DIV/0	#DIV/0
F10	2.91E-20	2.62E-23	3.31E-20	3.31E-20	3.48E-24	7.45E-06
F11	3.31E-20	#DIV/0	3.31E-20	3.31E-20	#DIV/0	#DIV/0
F12	7.96E-18	9.37E-10	7.96E-09	7.06E-13	8.25E-03	1.43E-06
F13	4.25E-16	7.38E-09	6.25E-19	2.24E-18	3.75E-02	2.32E-09
+/=/-	12/0/0	2009/2/1	12/0/0	12/0/0	10/2/0	10/2/0

### 4.4 Comparison with other improved ChOA algorithms

To further verify the superiority of the improvement strategy of the MChOA algorithm, the MChOA algorithm is compared with the EChOA algorithm in the literature [22], the SChOA algorithm in the literature [23] and the WChOA algorithm in the literature [24], and since it has been shown above that the MChOA algorithm can find the optimal value on most fixed-dimensional multi-peaked functions, this section selects F1-F13 as benchmark functions. The solution results of several improved ChOA algorithms are given in Table 8. and it can be seen that the MChOA algorithm has better performance, and it can be proved that the three improved strategies proposed in this paper are more suitable to be applied to the ChOA algorithm.

**Table 8:** Comparison with the improved ChOA algorithm for finding the best results.

	EChOA [22]		SChOA [23]		WChOA [24]		MChOA	
	Average	std	Average	std	Average	std	Average	std
F1	1.22E-36	6.33E-36	1.57E+03	5.68E+03	1.64E-26	3.00E-08	**0.00E+00**	**0.00E+00**
F2	6.57E-3	6.54E-23	1.02E+09	1.91E+10	1.00E-17	1.77E-06	**2.59E-173**	**3.74E-173**
F3	4.31E-19	1.59E-18	1.06E+03	2.26E+04	5.89E+00	1.11E-02	**7.14E-252**	**1.96E-253**
F4	2.57E-16	8.96E-16	1.22E+01	1.27E+00	1.83E-08	2.98E-04	**1.34E-159**	**2.32E-159**
F5	1.04E+01	8.21E-01	5.96E+05	1.01E+07	**1.01E-18**	**2.24E-06**	2.70E+01	3.56E-01
F6	1.11E+00	4.33E-01	1.01E+03	5.62E+03	5.03E-01	**9.86E-04**	**2.93E-01**	5.21E-02
F7	**1.09E-06**	**7.99E-07**	1.44E+00	5.77E+00	1.66E-03	4.41E-05	2.60E-05	1.49E-05
F8	—	—	-3.85E+03	1.80E+02	-1.17E+04	**2.41E+00**	**-1.25E+04**	1.25E+02
F9	1.41E-01	6.05E-01	4.40E+01	7.77E+01	1.56E-05	6.40E-03	**0.00E+00**	**0.00E+00**
F10	1.94E-14	3.63E-15	1.89E+00	1.72E+00	1.22E-15	5.05E-05	**4.44E-16**	**0.00E+00**
F11	3.10E-03	7.33E-03	2.10E+01	8.31E+01	0.00E+00	0.00E+00	**0.00E+00**	**0.00E+00**
F12	**7.45E-12**	**2.93E-12**	2.59E+06	3.31E+07	2.80E-02	2.67E-05	1.37E-02	4.82E-03
F13	**1.00E-10**	**2.87E-11**	1.61E+07	5.17E+07	8.42E-01	2.34E-02	2.78E-01	4.57E-02

## 5 Practical applications of MChOA

Production scheduling is a critical component of manufacturing production management, which involves planning and scheduling production workshops to optimize production plans and improve production efficiency. The main goal of production scheduling is to achieve timely completion of production plans, typically with the maximum completion time as the optimization objective, which is expressed as the objective function formula:

$$\begin{array}{c} F=\text{min}(\text{max}(Cj)1\leq j\leq n)i=1\cdots n\; \end{array}$$ (13)

Where n indicates the total number of work pieces, Indicates the completion time of work piece j.

To verify the performance of the comparison algorithm, this paper validates threeflexible job shop problem instances consisting of 8 workpieces with four processes, each on eight machines for the 8 × 4 × 8 partially flexible job shop scheduling problem; In order to validate the performance of the comparison algorithms, three examples of flexible job shop problems are validated in this paper, including a partially flexible job shop scheduling problem with 8 × 4 × 8, and a fully flexible job shop scheduling case with 10 × 4 × 10 and 15 × 4 × 10, where A × B × C stands for the number of workpieces, the number of processes, and the number of machines. The test results using the algorithm proposed in this paper are compared with those of the KACEM [29] method and the XIA [30] method. Table 9 shows that the proposed algorithm obtains the first rank in three cases, which can prove that the MChOA algorithm can be well applied in the field of shop floor scheduling, in order to verify the practicality of the algorithm, this paper carries out the verification of a practical application case.

**Table 9:** Results of each algorithm.

Example	Population	Methods Such as KACEM	Methods Such as XIA	MChOA	Computing Time
8X8	200	15	16	14	1.4
10X10	200	7	7	7	3.2
15X10	200	24	12	12	10.3

Company A was established in 1960 and now has a first-class high-tech machining center in the industry. It is mainly responsible for the production and machining of many types of aero-engine components, mainly magazines, blades, disc shafts, and many other types of parts, etc. As customer orders continue to increase, this poses a challenge to Company A’s workshop operation capacity. In this paper, we will use the production workshop of the company as a case study. The workshop comprises a total of 11 machines and is responsible for producing turbine discs for seven different types of aircraft engines. Each machining process can be performed using several machines, with varying machining times depending on the specific machine used. To simplify the presentation of machining data, we have compiled the information into Table 10.

**Table 10:** Workpiece processing information.

Workpiece	Work sequence	Process name	Processing time for each workpiece process on each machine										
			Lathe		boring machine	milling machine		Machining Center			grinding machine		Planer
			M1	M2	M3	M4	M5	M6	M7	M8	M9	M10	M11
1# Turbine Disc		turning	4	3				4	5	5			
		Milling				3	2	5	4	6			
		Planing											4
		Boring			5								
		Drill						4	6	5			
		Grinding									7	6	
2# Turbine Disc		Milling				5	6	9	8	7			
		Grinding									3	5	
		Boring			3								
		Drill						6	4	7			
		turning	5	6				7	8	7			
		Planing											5
3# Turbine Disc		Boring			3								
		Drill						5	4	6			
		turning	4	3				7	6	5			
		Planing											3
		Grinding									6	4	
		Milling				8	7	4	6	5			
4# Turbine Disc		Grinding									8	7	
		Planing											7
		Drill							4	6	5		
		turning	7	8					5	6	4		
		Milling					4	3	5	6	5		
		Boring			6								
5# Turbine Disc		Planing											3
		turning	9	8				6	7	5			
		Grinding									4	4	
		Milling				3	5	7	5	5			
		Boring			4								
		Drill						9	7	8			
6# Turbine Disc		turning	3	5				7	8	8			
		Boring			6								
		Milling				9	10	8	8	6			
		Grinding									8	6	
		Drill						3	4	3			
		Planing											4
7# Turbine Disc		Drill						5	6	5			
		Boring			7								
		Grinding									6	4	
		Milling				4	6	7	6	8			
		Planing											5
		turning	3	2				4	5	4			

**Fig 6:**
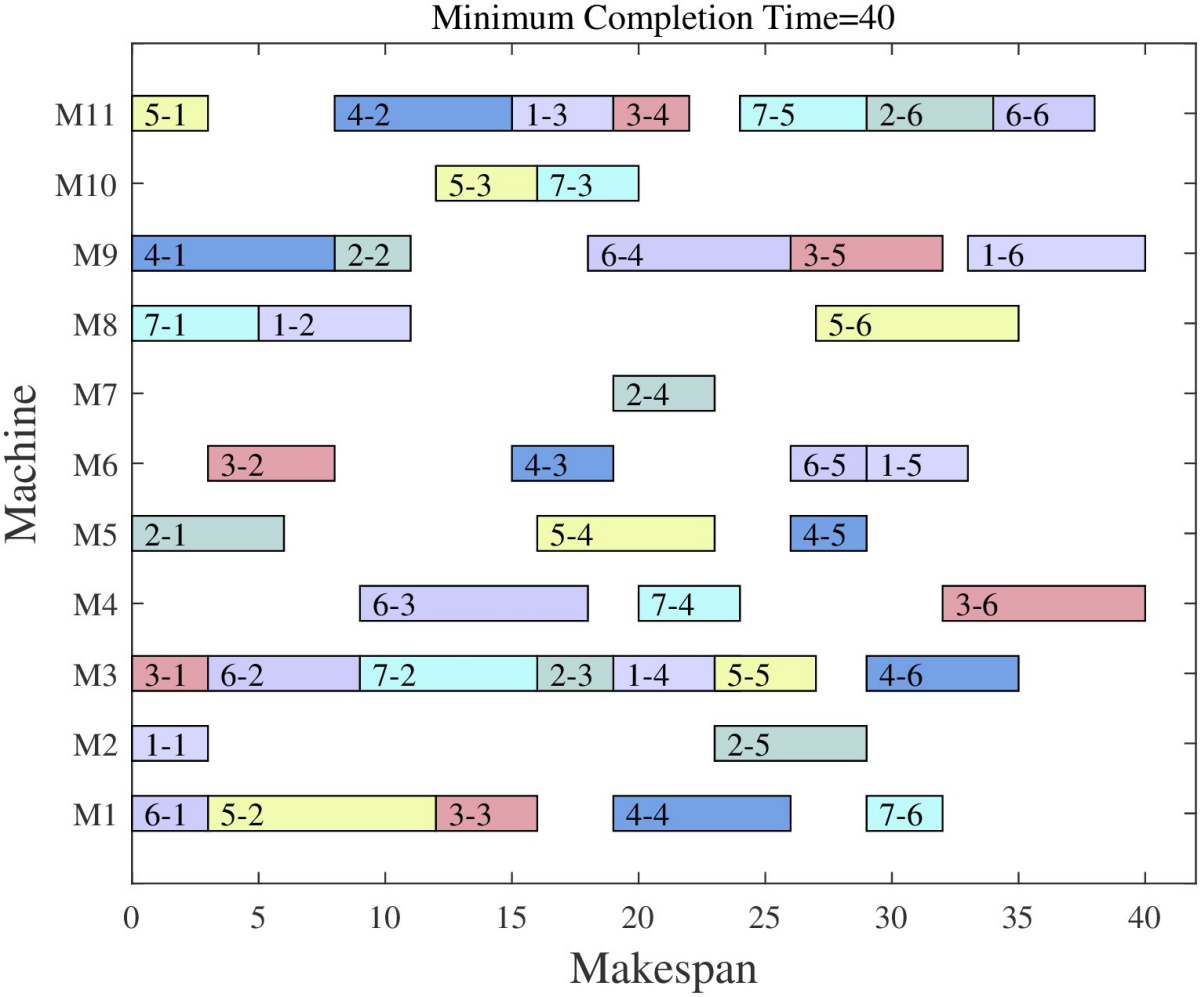
Gantt chart of workshop actual scheduling.

Based on the above data, the proposed algorithm was used to optimize the scheduling plan for this aero-engine workshop. Based on field research, the company’s current production decisions are made based on the experience of its employees, and the maximum completion time is 40h as indicated by the Gantt chart drawn after collating the production scheduling schedules provided by the company. The processing Gantt chart is shown in Fig 6.

**Fig 7:**
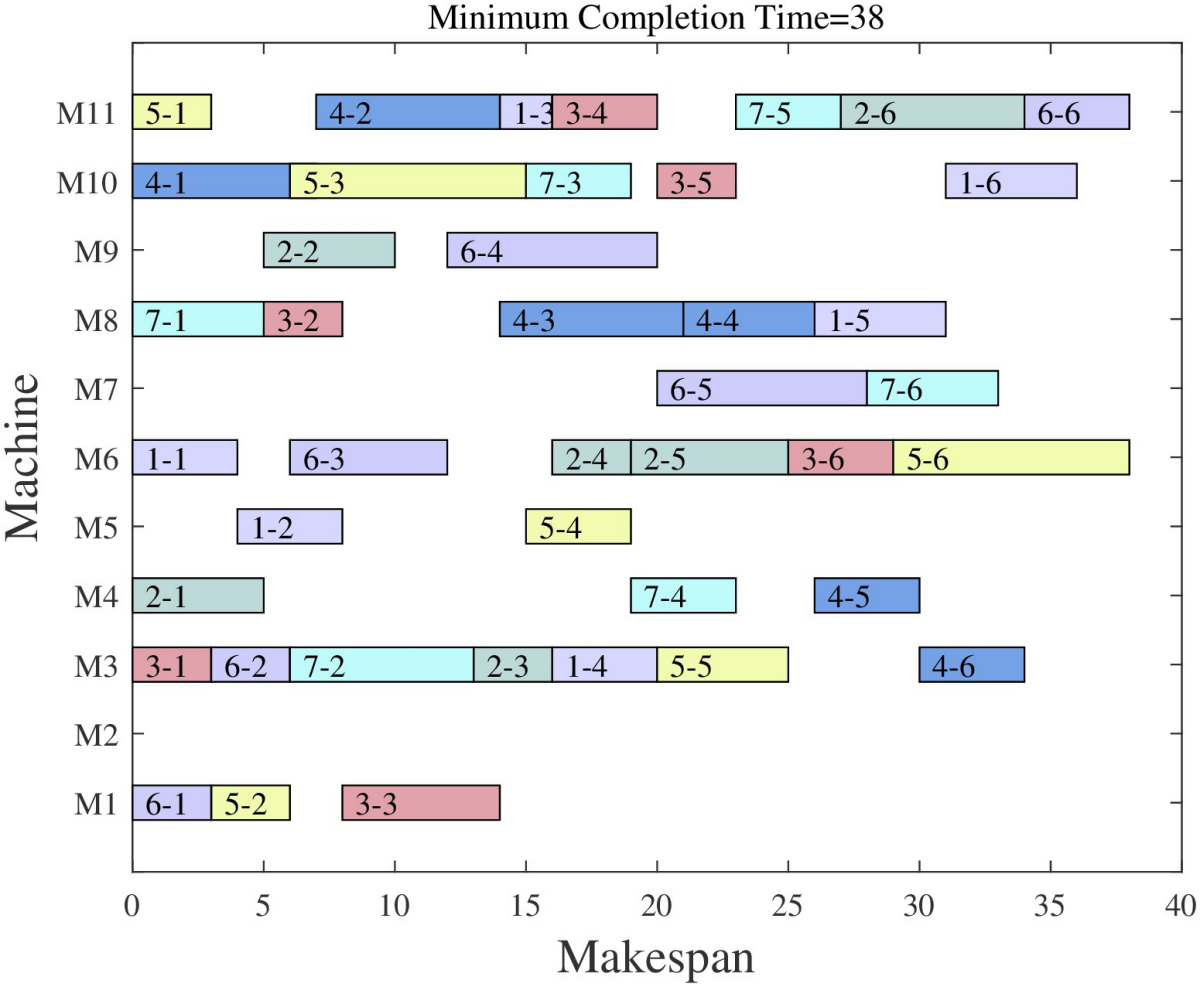
ChOA solution result.

**Fig 8:**
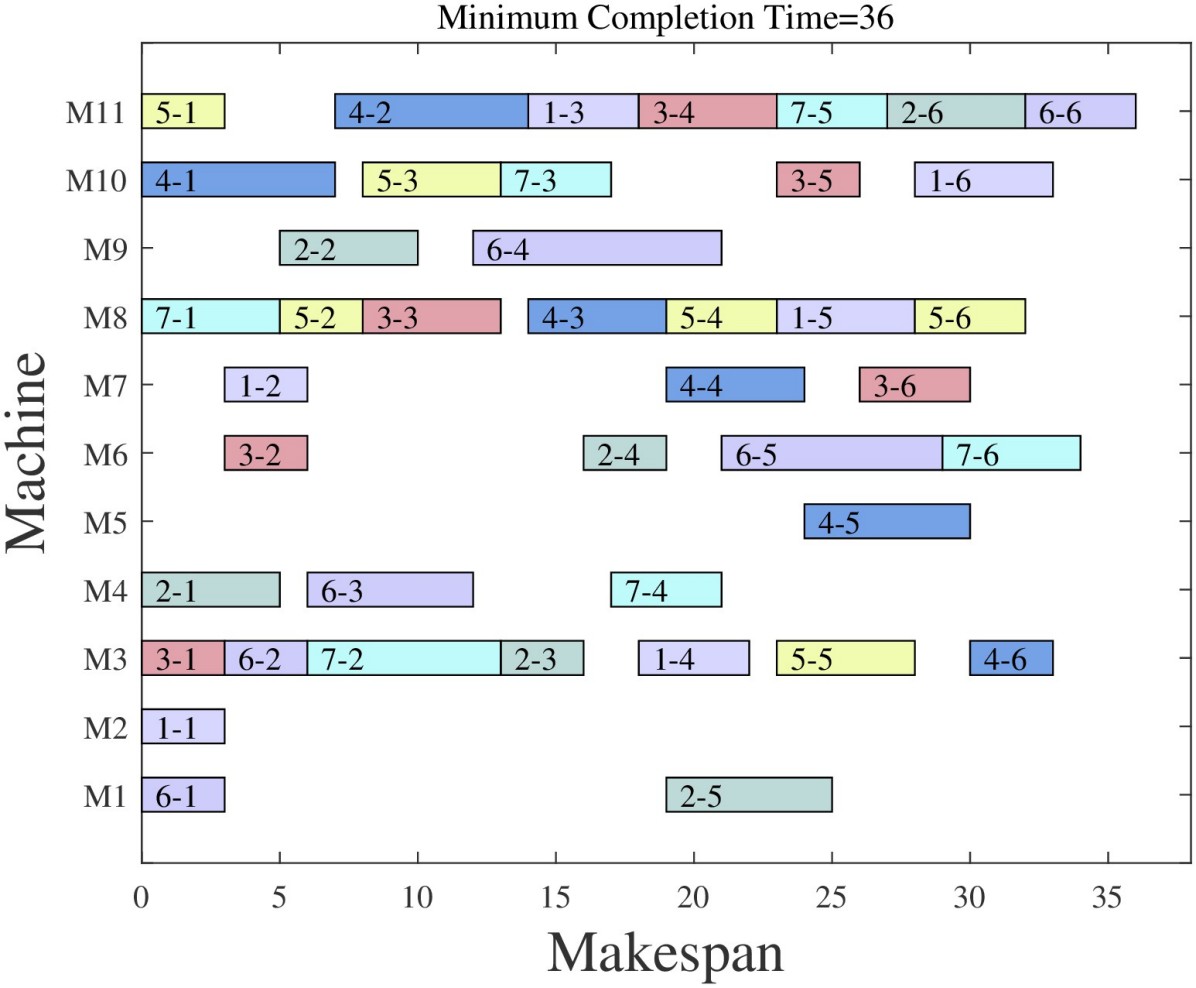
MChOA solution result.

Figs 7 and 8 give the ChOA algorithm and the solution scheme of MChOA algorithm for Company A. It can be seen that the ChOA algorithm has optimized the problem, and the algorithm designed in this paper is used for the optimization of the scheduling scheme of the aero-engine shop with a maximum completion time of 37 hours. The above results show that the reasonableness of the scheduling plan is improved after using MChOA to optimize the production scheduling of the company. This proves that the MChOA algorithm can also have good optimization performance on practical engineering problems.

## Concluding remarks

This paper introduces an improved chimp Optimization Algorithm based on the feeding strategy of manta rays(MChOA), aiming to enhance the performance of the basic chimp Optimization Algorithm (ChOA). The Latin hypercube method is employed to create an initial population with higher diversity and better search ability in the solution space. A convergence factor based on the tangent function is introduced to balance the global search and local exploitation capabilities, enhancing the algorithm’s early search ability and later development. During the position update process, the feeding strategy of manta rays is incorporated to prevent premature convergence. Experimental results indicate that, compared to many swarm-based algorithms on benchmark test functions, MChOA demonstrates outstanding performance. The paper also conducts a study on flexible job-shop scheduling problems using the same examples, comparing MChOA with other algorithms from the literature. The comparative results further improve the computational outcomes, validating the feasibility and effectiveness of the proposed approach. Finally, by optimizing the scheduling problem of an aerospace engine manufacturing plant, the optimized solution outperforms the actual scheduling plan of the aerospace company, highlighting the practical applicability of MChOA. However, the current proposed algorithm is temporarily limited to single-objective problems, and in future research, efforts will be made to further develop the ChOA algorithm for multi-objective problem applications.

## Supporting information

S1 File Data. (XLSX)

## References

[1] He DX, Zhou X, Wang Z, et al. Complex Network Community Mining: Genetic Algorithm Based on Clustering Fusion. Acta Automatica Sinica, 2010, 36(8):1160–1170. doi: 10.3724/SP.J.1004.2010.01160

[2] Kirkpatrick S, Gelatt CD, Vecchi MP. (1987) Optimization by Simulated Annealing, In: Fischler MA, Firschein O (Eds.), Readings in Computer Vision, San Francisco (CA), Morgan Kaufmann, 606–615.

[3] Parsopoulos KE, Vrahatis MN. Particle swarm optimization method for constrained optimization problems. Intelligent Technologies -Theory and Application: New Trends in Intelligent Technologies, 2002, 76(1): 214–220.

[4] Xiaoqian Shi, Qidong Chen, Jun Sun, et al. Adaptive distribution based quantum-behaved particle swarm optimization algorithm for engineering constrained optimization problem[J]. Journal of Computer Applications, 2020, 40(05): 1382–1388.

[5] Mirjalili S, Lewis A (2016) The Whale Optimization Algorithm. Advances in Engineering Software 95: 51–67. doi: 10.1016/j.advengsoft.2016.01.008

[6] ZHAO Y, PENG Z,R. Whale Optimization Algorithm with Chaotic Maps and Its Application in Finite Element Model Updating[J]. Journal of Lanzhou Jiaotong University, 2021, 40(01): 39–45.

[7] Bingol H, Alatas B. Chaos based optics inspired optimization algorithms as global solution search approach. Chaos, Solitons & Fractals. 2020;141: 110434. doi: 10.1016/j.chaos.2020.110434

[8] Bingol H, Alatas B. Chaos enhanced intelligent optimization-based novel deception detection system. Chaos, Solitons & Fractals. 2023;166: 112896. doi: 10.1016/j.chaos.2022.112896

[9] Izci D, Ekinci S, Kayri M, Eker E. A novel improved arithmetic optimization algorithm for optimal design of PID controlled and Bode’s ideal transfer function based automobile cruise control system. Evolving Systems. 2022;13: 453–468. doi: 10.1007/s12530-021-09402-4

[10] Eker E, Kayri M, Ekinci S, Izci D. A New Fusion of ASO with SA Algorithm and Its Applications to MLP Training and DC Motor Speed Control. Arab J Sci Eng. 2021;46: 3889–3911. doi: 10.1007/s13369-020-05228-5

[11] Izci D, Ekinci S, Zeynelgil HL, Hedley J. Fractional Order PID Design based on Novel Improved Slime Mould Algorithm. Electric Power Components and Systems. 2021;49: 901–918. doi: 10.1080/15325008.2022.2049650

[12] Ekinci S, Izci D, Abu Zitar R, Alsoud AR, Abualigah L. Development of Lévy flight-based reptile search algorithm with local search ability for power systems engineering design problems. Neural Comput & Applic. 2022;34: 20263–20283. doi: 10.1007/s00521-022-07575-w

[13] Ekinci S, Izci D, Kayri M. An Effective Controller Design Approach for Magnetic Levitation System Using Novel Improved Manta Ray Foraging Optimization. Arab J Sci Eng. 2022;47: 9673–9694. doi: 10.1007/s13369-021-06321-z

[14] Khishe M, Mosavi MR (2020) Chimp optimization algorithm. Expert Systems with Applications 149: 113338. doi: 10.1016/j.eswa.2020.113338 PMC9633109 36348736

[15] Zayed ME, Zhao J, Li W, et al. (2021) Predicting the performance of solar dish Stirling power plant using a hybrid random vector functional link/chimp optimization model. Solar Energy 222: 1–17. doi: 10.1016/j.solener.2021.03.087

[16] Kaur M SChoA: a newly fusion of sine and cosine with chimp optimization algorithm for HLS of datapaths in digital filters and engineering applications. Engineering with Computers 29.

[17] Liu C, H H,Q. Golden Sine Chimp Optimization Algorithm Integrating Multiple Strategies [J]. Acta Automatica Sinica: 1–14.

[18] He Q, Luo S,H. Chimp optimization algorthim based on hybrid improvement strategy and its mechanical application[J]. Contorl and Decision: 2023 1–11.

[19] Kaidi W, Khishe M, Mohammadi M (2022) Dynamic Levy Flight Chimp Optimization. Knowledge-Based Systems 235: 107625. doi: 10.1016/j.knosys.2021.107625

[20] Modified Chimp Optimization Algorithm Based On Classical Conjugate Gradient Methods—IOPscience Available from: https://iopscience.iop.org/article/10.1088/1742-6596/1963/1/012027.

[21] Yan C, Chen J, Ma Y (2019) Grey Wolf Optimization Algorithm with Improved Convergence Factor and Position Update Strategy, 2019 11th International Conference on Intelligent Human-Machine Systems and Cybernetics (IHMSC), 41–44.

[22] Sobol I. M., & Feinberg B. J. (1987). Minimum-variance unbiased estimation in simulation output analysis. ACM Transactions on Modeling and Computer Simulation (TOMACS), 3(2), 89–98.

[23] Zhao W, Zhang Z, Wang L (2020) Manta ray foraging optimization: An effective bio-inspired optimizer for engineering applications. Engineering Applications of Artificial Intelligence 87: 103300. doi: 10.1016/j.engappai.2019.103300

[24] Ma C, Huang H, Fan Q, et al. (2022) Grey wolf optimizer based on Aquila exploration method. Expert Systems with Applications 205: 117629. doi: 10.1016/j.eswa.2022.117629

[25] A Modified Particle Swarm Optimization with an Adaptive Acceleration Coefficients | Proceedings of the 2009 Asia-Pacific Conference on Information Processing—Volume 02 Available from: https://dl.acm.org/doi/10.1109/APCIP.2009.217.

[26] Tian D, Shi Z (2018) MPSO: Modified particle swarm optimization and its applications. Swarm and Evolutionary Computation 41: 49–68. doi: 10.1016/j.swevo.2018.01.011

[27] Faramarzi A, Heidarinejad M, Mirjalili S, et al. (2020) Marine Predators Algorithm: A nature-inspired metaheuristic. Expert Systems with Applications 152: 113377. doi: 10.1016/j.eswa.2020.113377

[28] Dehghani M, Hubálovský Š, Trojovský P (2021) Northern Goshawk Optimization: A New Swarm-Based Algorithm for Solving Optimization Problems. IEEE Access 9: 162059–162080. doi: 10.1109/ACCESS.2021.3133286

[29] Pezzella F.; Morganti G.; Ciaschetti G. A genetic algorithm for the Flexible Job-shop Scheduling Problem. Comput. Oper. Res. 2008, 35, 3202–3212. doi: 10.1016/j.cor.2007.02.014

[30] Xing L.-N.; Chen Y.-W.; Yang K.-W. An efficient search method for multi-objective flexible job shop scheduling problems. J. Intell. Manuf. 2009, 20, 283–293. doi: 10.1007/s10845-008-0216-z

